# Systemic inflammation and metabolic syndrome components in threshold/subthreshold posttraumatic stress disorder and food addiction in a Polish community sample

**DOI:** 10.1080/20008066.2025.2478792

**Published:** 2025-03-26

**Authors:** Monika M. Stojek, Marta Łukowska, Jagoda Różycka, Maryla Sokołowska, Joanna Zielińska, Ari Nowacki, Roksana Duszkiewicz, Anna Psurek, Vasiliki Michopoulos

**Affiliations:** a Trauma, Health and Eating (Thrive) Lab, Institute of Psychology, University of Silesia, Katowice, Poland; b Department of Psychiatry and Behavioral Sciences, Emory University School of Medicine, Atlanta, GA, USA; c Emotion Cognition Lab, Institute of Psychology, SWPS University, Katowice, Poland; d Faculty of Medicine, Medical University of Silesia, Katowice, Poland; e Faculty of Medicine, Academy of Silesia, Katowice, Poland; f Marie Skłodowska-Curie National Oncology Institute Research Institute, Gliwice, Poland

**Keywords:** Posttraumatic stress disorder (PTSD), food addiction, obesity, metabolic syndrome, inflammation, dysregulated eating, Trastorno de estrés postraumático (TEPT), adicción a la comida, obesidad, síndrome metabólico, inflamación, alimentación desregulada

## Abstract

**Background:** Posttraumatic stress disorder (PTSD) is associated with metabolic syndrome and various addictive behaviours. Food addiction (FA) is associated with obesity, and individuals with PTSD have higher rates of FA than those without. It is unclear whether addictive-like eating patterns contribute to the metabolic dysfunction in PTSD.

**Objective:** We examined the relative contributions of PTSD, FA, and sex – as well as their interactive effects – to the systemic inflammation (CRP) and metabolic syndrome components (MetS: waist circumference, glucose, triglycerides, HDL cholesterol, insulin) in a general population of the Upper Silesia region in Poland.

**Method:** *N* = 187 participants (52.7% women) completed Yale Food Addiction Scale 2.0 (FA symptoms count), Clinician-Administered PTSD Scale for DSM-5 (CAPS-5) semi-structured interview (PTSD or other trauma and stressor-related disorder (OTSR) diagnosis presence), anthropometric assessment, and phlebotomy in a fasted state.

**Results:** A series of hierarchical linear regressions indicated that greater number of FA symptoms had a significant effect on greater waist circumference, while PTSD/OTSR diagnosis had a significant effect on higher insulin levels. Sex did not moderate these relationships.

**Conclusions:** It appears that dysregulated eating patterns are associated with greater abdominal obesity, but not with metabolic dysfunction. PTSD/OTSR, but not FA, contributes to greater insulin levels. The average metabolic indices were within normal limits reflecting a non-clinical nature of the sample. Future longitudinal studies should examine whether detection of and intervention for PTSD/OTSR symptoms may be a strategy for preventing progression of metabolic dysfunction.

## HIGHLIGHTS

- In a general Polish population sample, food addiction symptoms are associated with abdominal obesity but not with other studied metabolic syndrome components (i.e. glucose, triglycerides, HDL cholesterol), insulin, or systemic inflammation.
- Threshold and subthreshold PTSD was associated with greater insulin levels but not with systemic inflammation, abdominal obesity or other metabolic syndrome components.
- In this non-treatment-seeking community sample, excess weight is uniquely related to dysregulated eating, while greater insulin levels are uniquely associated with threshold and subthreshold PTSD diagnosis but not eating dysregulation.

## Abstract (es)

**Antecedentes:** El trastorno de estrés postraumático (TEPT) está asociado con el síndrome metabólico y diversas conductas adictivas. La adicción a la comida (FA en sus siglas en inglés) está asociada con la obesidad, y las personas con TEPT tienen tasas más altas de FA que las que no la padecen. No está claro si los patrones de alimentación similares a la adicción contribuyen a la disfunción metabólica en el TEPT.

**Objetivo:** Examinamos las contribuciones relativas del TEPT, la FA y el sexo, así como sus efectos interactivos, a los componentes de la inflamación sistémica (PCR) y del síndrome metabólico (MetS: circunferencia de la cintura, glucosa, triglicéridos, colesterol HDL, insulina) en una población general de la región de Alta Silesia en Polonia.

**Método:** *N* = 187 participantes (52.7% mujeres) completaron la Escala de Adicción a la Comida de Yale 2.0 (recuento de síntomas de FA), la Escala de TEPT Administrada por el Clínico para el DSM-5 (CAPS-5), entrevista semiestructurada (presencia de diagnóstico de TEPT u otro trastorno relacionado con trauma y estrés [OTSR en sus siglas en inglés]), evaluación antropométrica y flebotomía en estado de ayuno.

**Resultados:** Una serie de regresiones lineales jerárquicas indicó que un mayor número de síntomas de FA tuvo un efecto significativo en una mayor circunferencia de cintura, mientras que el diagnóstico de TEPT/OTSR tuvo un efecto significativo en niveles más altos de insulina. El sexo no moderó estas relaciones.

**Conclusiones:** Parece que los patrones de alimentación desregulados están asociados con una mayor obesidad abdominal, pero no con disfunción metabólica. El TEPT/OTSR, pero no la FA, contribuyen a una mayor respuesta inflamatoria y mayores niveles de insulina. Los índices metabólicos promedio estuvieron dentro de los límites normales, lo que refleja una naturaleza no clínica de la muestra. Los estudios longitudinales futuros deberían examinar si la detección y la intervención de los síntomas de TEPT/OTSR pueden ser una estrategia para prevenir la progresión de la disfunción metabólica.

Posttraumatic stress disorder (PTSD) is a heterogeneous stress-related psychiatric disorder that develops following trauma exposure (American Psychiatric Association, 2013; Kessler et al., 1995). Apart from psychosocial impairment, PTSD is associated with worsened physical health (Pacella et al., 2013). Individuals with PTSD have been found to be at higher risk for metabolic syndrome (MetS) (Boscarino, 2008; Kubzansky et al., 2009; Xue et al., 2012) – a constellation of health risk factors including central obesity, hypertension, elevated glucose and triglycerides, and low high-density lipoprotein (HDL) cholesterol – which in turn is related to the risk of developing cardiovascular disease (Mottillo et al., 2010). PTSD is also associated with elevated insulin levels, contributing to metabolic dysfunctions (Michopoulos et al., 2016). A systematic review and meta-analysis of over 9,000 participants with PTSD (and over 6000 matched controls) found that people with PTSD had almost double the risk of having MetS compared to matched controls (Rosenbaum et al., 2015).

The putative changes in inflammatory signalling in PTSD are suggested to stem from alterations in glucocorticoid sensitivity and sympathetic nervous system activity (Michopoulos et al., 2017). They may contribute to not only behavioural symptoms of PTSD but also the accompanying manifestations of metabolic dysfunction (Michopoulos et al., 2016). Elevated inflammation co-occurs with increased sympathetic tone in MetS as the noradrenergic system also stimulates the innate immune response (Michopoulos et al., 2016). Increased levels of the pro-inflammatory marker C-reactive protein (CRP) have been observed in individuals with PTSD (Michopoulos et al., 2015; Spitzer et al., 2010). In fact, a single nucleotide polymorphism within the CRP gene (*rs1130864*) has been associated with increased susceptibility to a PTSD diagnosis in a sample of highly traumatized individuals (Michopoulos et al., 2015). Overall, there is consistent evidence that PTSD symptoms are associated with chronic systemic inflammation. Moreover, it appears that inflammation may be the common mechanism behind PTSD symptoms expression and associated metabolic dysfunction.

Individuals with PTSD are at higher risk of developing addictive behaviours (Debell et al., 2014; Konkolÿ Thege et al., 2017; Lawson et al., 2013). One addictive phenotype that has been found to positively associate with PTSD symptoms is food addiction (FA) (Masheb et al., 2018; Mitchell & Wolf, 2016; Stojek et al., 2021). FA is a dysregulated eating phenotype encompassing behaviours analogous to substance use disorders, including persistent attempts to cut out high-calorie food, experiencing social problems related to consumption, or developing tolerance (Gearhardt et al., 2009; Gearhardt et al., 2016). FA is not a DSM-5 or ICD-11 diagnosis, but it has emerged as a useful construct in obesity research (Cassin & Sockalingam, 2021).

Most studies indicate a relationship between FA severity and body mass index (BMI) (Meule & Gearhardt, 2019; Schulte & Gearhardt, 2018). Associations of FA with MetS and inflammatory biomarkers have been more scarce and equivocal. With regard to waist circumference, FA has been associated with greater waist circumference in college students (Şengör & Gezer, 2019) and in the general population (Pedram et al., 2013), but not in patients with obesity (Kiyici et al., 2020) or type 2 diabetes mellitus (T2DM) (Nicolau et al., 2020). With regard to fasting glucose, no study to date examined the association between FA and glucose levels in the general population, while findings in the clinical populations indicate positive association between FA and fasting glucose in patients with T2DM (Nicolau et al., 2020), but a negative association in patients with obesity (Nicolau et al., 2020). Regarding triglycerides, studies in general populations found some sex effects in the positive association between FA and higher triglyceride levels (Nelder et al., 2018), but no associations in those with obesity (Kiyici et al., 2020) or T2DM (Nicolau et al., 2020). Regarding HDL cholesterol, only one study indicated an inverse association between FA and HDL cholesterol levels in men (but not women) (Nelder et al., 2018), while two found no associations inpatients with T2DM (Nicolau et al., 2020) or obesity (Kiyici et al., 2020). Regarding fasting insulin, one study found that FA was positively associated with insulin levels (Lopez-Aguilar et al., 2018), while another failed to do so (Kiyici et al., 2020). No study examined the relationship between CRP and FA.

Given that PTSD diagnosis is positively associated with FA symptoms (Masheb et al., 2018; Mitchell & Wolf, 2016; Stojek et al., 2021), it is likely that in a subset of individuals with PTSD, food serves a similar function to substances of abuse. The findings on the relationship between FA and MetS are in nascency and inconclusive. Therefore, it would be helpful to examine the whether there is a link between FA and the metabolic dysfunction associated with PTSD in order to better address health problems among people with PTSD. Importantly, there are sex differences in the prevalence of PTSD, obesity, and FA. Women are twice as likely to develop PTSD following a traumatic event (Steven Betts et al., 2013): lifetime prevalence stands at 10–12% in women, compared to 5–6% in men (Olff, 2017). In Poland, rates of overweight/obesity vary by sex, with more men than women carrying excess weight (GUS, 2016). Some (Meule & Gearhardt, 2019; Pursey et al., 2014), but not all (Bourdier et al., 2018; Stojek et al., 2021), studies report differences in FA prevalence based on sex. Therefore, it is important to include sex as a variable in studies on PTSD, eating, and health.

In the current study, we sought to examine the effect of PTSD presence, FA symptoms severity, and their interaction on inflammation and four of five elements of the MetS: waist circumference (WC), fasting plasma glucose, triglycerides, and HDL cholesterol (HDLchol). Given the biological link between PTSD and increased insulin levels (Michopoulos et al., 2016), fasting insulin levels were also included as an outcome variable. We included sex as a predictor of interest to account for potential sex differences (Haering et al., 2024). We hypothesized that there would be a significant effect of PTSD diagnosis on the four MetS components, insulin, and systemic inflammation, such that individuals with PTSD will have significantly higher WC, glucose, triglycerides, and HDLchol (MetS components) as well as insulin and CRP compared to those without the diagnosis. We also hypothesized that there would be an effect of FA on the four MetS components, insulin and inflammation but given mixed findings, we did not make directional hypotheses. Finally, we hypothesized that there would be a significant effect of the PTSD and FA comorbidity on the four MetS components, insulin and inflammation. We included a three-way interaction of PTSD, FA symptoms and sex to explore potential sex effects but made no hypotheses regarding these effects.

## 1. Methods

### 1.1. Participants and procedure

Participants were recruited from the Upper Silesia region of Poland. Recruitment strategies included distributing information about the study via university newsletter, leaflets, posters, project’s social media and website, and paid advertisements on Facebook. Additionally, participants were recruited via an external pollster company (Nationwide Research Panel Ariadna; https://panelariadna.pl/). Inclusion criteria were age 18–55 and living in the Silesia region (to ensure the ability to attend the in-person laboratory session in Katowice). Exclusion criteria were current pregnancy or being less than 9 months since the last child delivery, and active psychosis. As part of a larger data collection, participants first completed online questionnaires to assess demographics as well as food addiction (FA) and PTSD symptoms (Stojek et al., under review). To determine laboratory session eligibility, a telephone screen was conducted to assess pregnancy/postpartum status, and psychosis symptoms; if eligible, participants were invited for the in-person laboratory session.

The laboratory session took place at the Institute of Psychology at the University of Silesia in Katowice and lasted approximately 3–4 h. The session included informed consent, anthropometric measurement, medication interview, a semi-structured CAPS interview (see 1.2. Measures), and several computer-based questionnaires and tasks. The computer-based questionnaires and tasks measured relative reinforcing value to food, attention bias to food and socially threatening stimuli, as well as liking of certain food items (during this task, skin conductivity was also measured). The computer-based questionnaires and tasks are not described here as these variables were not of interest in the current analyses. The anthropometric data were collected at the beginning of the laboratory session, immediately following the informed consent. The CAPS-5 interview was administered by a trained master’s or doctoral-level trainees supervised by the study PI (MMS) and another study clinical psychologist (JR). Following the interview, participants underwent a relaxation mood induction to alleviate the potential negative affect elicited by the content of the interview. The order of the clinical interview and the computer-based questionnaires and tasks was counterbalanced across participants, and participants were required to take a 15-minute break between the interview block (diagnostic interview + relaxation procedure) and computer-based block. At the end of the laboratory session, participants were instructed to attend a phlebotomy session in a fasting state within 5 days. Participants received a voucher to their local branch of the commercial phlebotomy laboratory (DIAGNOSTYKA; https://english.diag.pl/pacjent/) as well as written instructions to fast for 12 h before attending the phlebotomy session. High-sensitivity CRP, plasma glucose, serum insulin, triglycerides and cholesterol levels were obtained from 6 mL of blood draw.

Participants received monetary compensation of up to 250 PLN in three instalments for completing the laboratory session, a diagnostic interview and phlebotomy (they had the option to dissent from each of these study elements). The study was approved by the Bioethics Board of the Silesian Doctor’s Chamber in Katowice, Poland (nr 23/2022). Participation in this study was voluntary. The study was conducted in accordance with the Declaration of Helsinki.

### 1.2. Measures

#### 1.2.1. Psychological symptoms

*The Yale Food Addiction Scale* (YFAS 2.0) (Gearhardt et al., 2016); Polish adaptation (Buczny et al., 2017), assesses eleven symptoms of food addiction (FA) experienced within the previous 12 months. The symptoms align with the DSM-5 criteria for substance use disorder. The scale comprises 35 items in the form of first-person affirmative sentences, which participants rated on an 8-point Likert scale, ranging from 0 ‘Never’ to 7 ‘Daily’. The recommended scoring algorithm was applied to determine the FA symptom severity (https://sites.lsa.umich.edu/fastlab/yale-food-addiction-scale/). The internal consistency of the YFAS in our sample was alfa = .96 (95% CI = .95-.97).

*Clinician-Administered PTSD Scale for DSM-5* (CAPS-5) (Weathers et al., 2018) and *Clinician-Administered PTSD Scale for DSM-5 Revised* (CAPS-5-R) (Jackson et al., 2025) is a semi-structured diagnostic interview for assessment of PTSD symptoms presence and severity over the past month. The symptoms assessed with this interview correspond to the clinical criteria for PTSD in the DSM-5 (American Psychiatric Association, 2013) and include criterion A (presence of a past traumatic event), criterion B (re-experiencing), criterion C (avoidance), criterion D (alterations in mood and cognition) and criterion E (hyperarousal). The interview scoring algorithm allows for determining the presence of the PTSD diagnosis and yields a severity score based on clinician’s rating of each symptom. The first 20 participants were administered the CAPS-5, while the remainder of the participants were administered the CAPS-5-R (Jackson et al., 2025) – a version with improved administration and scoring (CAPS-5-R was not yet available at the beginning of the study). Both CAPS-5 and CAPS-5-R use the same DSM-5 PTSD criteria to determine the PTSD diagnosis presence. Validation of the CAPS-5-R demonstrated good to excellent alternate forms reliability with the CAPS-5 for determining PTSD diagnosis (ĸs = .79–.93) (Jackson et al., 2025).

In this study, we used only the diagnosis presence/absence criterion (as opposed to the continuous severity scoring). This was done to maximize the number of participants included in the analyses. As establishing whether the participants met PTSD diagnosis was the main goal of the interview, a procedure was implemented initially wherein the interview was discontinued if the participants did not meet any of the criteria B–E following meeting criterion A. This procedure was implemented to reduce participant burden during the laboratory session. The discontinuation procedure was implemented with 89 (of the total 212) participants, of whom 23 did not complete the full CAPS because they did not meet one of the B–E criteria. Starting with participant 90, all participants who met criterion A were administered the full CAPS.

Participants who met all the PTSD criteria were classified as ‘PTSD present’ and participants who met criterion A plus 3 out of 4 PTSD criteria were classified as ‘Other Trauma and Stressor-Related Disorder Present’ (OTSR), signifying subthreshold PTSD. Previous studies have indicated that subthreshold PTSD has comparably detrimental effect on functioning as threshold PTSD (El-Gabalawy et al., 2018), therefore the PTSD group consists of individuals with threshold and subthreshold PTSD symptoms.

The PTSD Checklist for DSM-5 (PCL-5) (Blevins et al., 2015; Ogińska-Bulik et al., 2018) is a 20-items self-report measure that evaluates the severity of PTSD symptoms in the past month in accordance with the DSM-5 criteria. Answers are marked on a 5-point Likert scale (from 0 – ‘Not at all’ to 4 – ‘Extremely’). The total symptom score ranges from 0 to 80. The internal consistency in our sample was α = .938.

#### 1.2.2. Anthropometrics

*Waist circumference* (cm) was measured using a tape measure at a midpoint point between the bottom of the rib cage and the top of the hips, just above the belly button. The measurement was taken in triplicate, the reported values are the average of the three measurements. Additionally, hips circumference was measured but was not used in the current analyses.

*Body mass index* was calculated using the standard equation (weight [kg]/squared height [m]). Height (cm) was measured in triplicate using a Chorder HM 200P stadiometer, and the average of the three measurements was reported. Weight (kg) was taken using the Tanita Inner Scan V BC-601 scale; participants were weighed barefoot and wearing light clothing. Of note, BMI was only used as a control variable in the regression models with biomarkers as outcome variables.

#### 1.2.3. Biomarkers

*High-sensitivity plasma C-reactive protein (hs-CRP)* was used to measure the level of systemic inflammation. hs-CRP was measured with a Roche Cobas C 311 analyzer and the Alinity c CRP Vario Reagent Kit. The assays did not detect values less than 0.15 mg/L (Roche Cobas) for eight participants (these values were recoded to 0.14 mg/L for the analyses) and less than 0.4 mg/L (Alinity) for three participants (these values were recoded to 0.39 for the analyses). Standard calibration was performed at every change of the reagent serial number or more often if quality control procedures indicated the need for additional calibration.

*Plasma glucose and serum insulin* were collected in a fasted state (at least 12 h since the last meal). Glucose and insulin concentrations were measured with the Alinity c Glucose Reagent Kit and the Alinity i Insulin Reagent Kit. Standard calibration was performed at every change of the reagent serial number or more often if quality control procedures indicated the need for additional calibration. Glucose concentrations are reported in mg/dL and insulin in µIU/mL.

*Triglycerides and high-density lipoprotein (HDL) cholesterol* measurements were conducted in a fasted state and concentrations are reported in mg/dL. Triglycerides and HDLchol concentrations were measured with the Alinity c Triglyceride2 and Alinity c Cholesterol2 Kits. Standard calibration was performed at every change of the reagent serial number or more often if quality control procedures indicated the need for additional calibration.

#### 1.2.4. Control variables

*Medications*. Participants were asked by the experimenter about the names of the medications they are currently taking from suggested categories: psychiatric medications, hypertension medications, diabetes medications, hormonal medications, supplements, and other drugs. The medications were classified using Anatomical Therapeutic Chemical Classification prepared by WHO. Medication use was coded as a binary variable indicating whether the person takes it or not. In the biomarkers models, the following medications were used as control variables: all reported diabetes medications (for glucose and insulin), all reported dyslipidemia medications (for triglycerides and HDL cholesterol), second generation antihistamines and all other antihistamines (for CRP).

*Time*. Study design required that participants completed the self-report measures of FA and PTSD symptoms prior to the laboratory session. Therefore, the number of days elapsed between self-report measures and laboratory session was used as one of the control variables to adjust for the potential effect of the time gap.

*Demographics*. Gender was assessed using the question: ‘What gender are you?’ Participants could answer: female, male, non-binary, other. In the phone screen, participants identified sex assigned at birth binary labels (female/male). Since sex was used as a predictor variable, individuals who identified their gender as non-binary were excluded from the current analyses (*n* = 1).

*Education*. Participants indicated education by selecting: primary, junior high school, vocational, high school, post-secondary, bachelor’s, master’s, doctoral. We further aggregated the above options into 5 categories: primary, vocational, secondary, post-secondary, and higher.

*Smoking.* Participants indicated their cigarette smoking status: current smoker, past smoker but had not smoked in the past 30 days, non-smoker.

*Age.* Participants indicated their age at the time of the session.

### 1.3. Analyses

Outliers were examined for the outcome variables and adjusted to fall 1.5 times the interquartile range below and above the 25th and 75th percentile (Behrens, 1997). This strategy minimizes the influence of outliers on the characteristics of the distribution, minimally changes the distribution, and avoids potential bias of removing outliers altogether. Missing data were examined and if the visual analysis of missing data patterns indicated data were missing at random (MAR), multiple imputation was applied (Sterne et al., 2009). Data were examined for normality; hs-CRP, triglycerides, and insulin were log-transformed due to a positive skew. To adjust for the number of biomarkers family tests, a Bonferroni correction was applied, and the statistical significance level was set to *p* = .010. All analyses were conducted using the IBM SPSS software, version 29.

To test the hypotheses, we conducted one hierarchical linear regression with CRP as an outcome variable and five hierarchical linear regressions related to MetS components: WC, glucose, triglycerides, HDLchol, and insulin. In the model with WC, in step 1, we entered variables for which we wanted to adjust the model, i.e. age, education level, alcohol use severity, smoking status, time between session. In step 2, we entered sex; in step 3, the number of FA symptoms; in step 4, PTSD symptom severity; in step 5, PTSD/OTSR diagnosis; in step 6, the interaction of PTSD/OTSR and FA symptoms; in step 7, the interaction of PTSD/OTSR, FA symptoms, and sex. The order of variables in the model was selected based on theoretical assumptions and past literature. Sex was entered as the first predictor because there are sex differences in obesity rates. FA was entered next because there is consistent body of research indicating its relationship to obesity, including WC in the general sample.

In all other models, we adjusted for different classes of medications. In step 1, we included variables for which we wanted to statistically adjust the model, including age, education level, smoking status, alcohol use problems, BMI, and time between sessions, and different classes of medications depending on the outcome variable: antihistamines for CRP, diabetes drugs for glucose and insulin, and dyslipidemia drugs for triglycerides and HDLchol. In step 2, we included sex (as there are sex differences in health outcomes). In step 3, we entered PTSD symptom severity and in step 4, we entered PTSD/OTSR presence (as PTSD symptoms have been consistently associated with inflammation and other biomarkers of health). In step 5, we entered FA symptom number. The remainder of the steps were identical to the WC model described above.

## 2. Results

### 2.1. Descriptive statistics

A total of 212 participants consented to the laboratory session. Of those, 23 participants did not have sufficient data to determine whether they met criteria for PTSD or OTSR based on the CAPS-5 (see 1. Methods). Since these data were not missing at random, those cases were removed from the analyses. Additionally, one participant did not undergo WC measurement, nor did (s)he attend phlebotomy. One participant identified their gender as non-binary and was excluded from the analyses. Therefore, a total of 187 participants were included in the final dataset. Of those, 9.6% were missing hsCRP, 8% were missing insulin, 7% were missing glucose, and 6.4% were missing triglycerides results; following the multiple imputation procedure, 176 participants’ biomarker data were analysed. All biomarkers results are reported on the pooled data from five iterations of the imputation.

Descriptive statistics for the full sample (*N* = 187) are reported in Table 1. The average age of participants was 30 years old (SD = 10; range = 18–55). Participants were on average slightly overweight and on average low-risk alcohol drinkers. Participants with the PTSD/OTSR diagnosis (*n* = 77) reported significantly more FA symptoms and significantly greater severity of PTSD symptoms.

**Table 1.** Descriptive statistics of key variables and control variables in the total sample (*N* = 187) as well as subsamples of participants with (*n* = 77) and without PTSD/OTSR diagnosis (*n* = 110).

	Whole sample (*N* = 187)	PTSD/OTSR diagnosis (*n* = 77)	No PTSD diagnosis (*n* = 110)	Comparison
Age *M* (SD)	30.73 (10.17)	30.38 (10.52)	30.97 (9.96)	*t* = .39, *p* = .347
AUDIT *M* (SD)	7.34 (5.88)	7.65 (6.12)	7.12 (5.73)	*t* = −.61, *p* = .272
Body Mass Index *M* (SD)	26.40 (5.97)	26.72 (6.31)	26.19 (5.73)	*t* = −.60, *p* = .275
Smoker *n* (%) positive	43 (23%)	21 (27.3%)	22 (20.0%)	*χ^2^* *=* 3.27, *p* = .195
Education level *n* (%)				*χ^2^* *=* 7.49, *p* = .112
Primary	2 (1.1%)	2 (2.6%)	0	
Vocational	2 (1.1%)	0	2 (1.8%)	
Secondary	80 (42.6%)	35 (45.5%)	45 (40.5%)	
Post-secondary	41 (21.9%)	20 (26%)	21 (19.1%)	
Higher	62 (33.2%)	20 (26%)	42 (38.2%)	
Type of medication *n* (%)				
All diabetes med.	9 (4.8%)	6 (7.8%)	3 (2.7%)	*χ^2^* = 2.54, *p* = .111
All dyslipidemia med.	3 (1.6%)	2 (2.6%)	1 (0.9%)	*χ^2 ^= *.82, *p* = .366
Antihistamines, 2nd gen.	8 (4.3%)	4 (5.2%)	4 (3.6%)	*χ^2 ^= *.27, *p* = .604
All other antihistamines	6 (3.2%)	2 (2.6%)	4 (3.6%)	*χ^2 ^*= .16, *p* = .692
Time *M* (SD)	69.07 (46.99)	61.97 (39.75)	74.04 (51.05)	*t* = 1.74, *p* = .042
Sex *n* (%) women	98 (52.4%)	44 (57.1%)	54 (49.1%)	*χ^2^* *=* 1.18, *p* = .278
PCL-5 *M* (SD)	35.15 (18.14)	28.59 (17.32)	44.52 (14.96)	*t* = −6.54, *p* < .001
Food addiction symptom count	3.67 (3.37)	4.56 (3.50)	3.05 (3.15)	*t* = −3.09, *p* = .001
Waist circumference (cm)	91.82 (17.80)	91.74 (19.12)	91.88 (16.90)	*t* = .06, *p* = .478
Fasting plasma glucose (mg/dL)	89.19 (8.90)	89.77 (9.04)	88.76 (8.81)	*t* = −.73, *p* = .465
Triglycerides (mg/dL)	100.03 (44.62)	107.74 (46.51)	94.44 (42.55)	*t* = −2.03, *p* = .042
HDL Cholesterol (mg/dL)	55.63 (13.54)	56.79 (14.88)	54.79 (12.49)	*t* = −.96, *p* = .337
Fasting insulin (µlU/L)	8.32 (4.14)	9.02 (4.30)	7.81 (3.96)	*t* = −1.89, *p* = .059
High-sensitivity C-reactive protein	1.81 (1.93)	2.06 (2.06)	1.62 (1.82)	*t* = −1.64, *p* = .101

Note: AUDIT = severity of alcohol use-related problems according to the Alcohol Use Disorder Identification Test; PCL-5 = PTSD symptom severity according to the PTSD Symptom Checklist for DSM-5.

### 2.2. Regressions

The coefficients for the model with waist circumference as the outcome variable (*N* = 187) are reported in Table 2. The addition of the sum of FA symptoms variable in step 3 significantly improved the model fit (*F_change _*= 16.785, *p* < .001). The final model accounted for 37.8% in the waist circumference variance, with only male sex and FA symptoms sum contributing significant variance to the model in the final step.

**Table 2.** Hierarchical linear regression model predicting variance in waist circumference (*N* = 187).

	*R*	*R*^2^	*t*	*p*
Step 1	.477	.228		
Age			6.030	<.001
Education level			.279	.781
AUDIT			1.384	.166
Smoking status			.564	.573
Time difference			−1.273	.203
Step 2	0.560	0.314		
Sex			−4.762	<.001
Step 3	0.611	0.373		
Sex			−5.378	<.001
FA			4.097	<.001
Step 4	0.611	0.373		
Sex			−5.332	<.001
FA			4.004	<.001
PCL-5			−.180	.857
Step 5	0.611	0.373		
Sex			−5.303	<.001
FA			4.016	<.001
PCL-5			−.001	.999
PTSD/OTSR diagnosis			−.420	.675
Step 6	0.612	0.374		
Sex			−5.302	<.001
FA			3.148	.002
PCL-5			−.025	.980
PTSD/OTSR			−.003	.998
FA × PTSD/OTSR			−.375	.708
Step 7	0.614	0.378		
Sex			−5.045	<.001
FA			3.216	.001
PCL-5			−.031	.975
PTSD/OTSR			.050	.961
FA × PTSD/OTSR			−.842	.400
FA × PTSD/OTSR × sex			.995	.320

Note: AUDIT = severity of alcohol use-related problems according to the Alcohol Use Disorder Identification Test; FA = sum of food addiction symptoms on the Yale Food Addiction Scale (YFAS); PCL-5 = PTSD symptom severity according to the PTSD Symptom Checklist for DSM-5; PTSD/OTSR = post-traumatic stress disorder or other trauma and stressor-related (i.e. criterion A + 3 out of 4 PTSD symptoms) diagnosis according to the CAPS-5 interview.

The coefficients for the models with the remainder of MetS components are reported in Table 3 (*N* = 176 after multiple imputations of missing values). All significance levels are reported at the significance level set by the Bonferroni correction (*p* < .010) to control for biomarkers family-wise error. For the model with systemic inflammation as the outcome variable, the final model accounted for 35.8% of the variance. Only female sex was a significant predictor of greater systemic inflammation once all variables were included in the model. In the model predicting fasting glucose level, none of the variables of interest emerged as statistically significant predictors. In the model with triglyceride levels as the outcome variable, the final model accounted for 26.9% of the variance. None of the variables of interest emerged as significant predictors of triglyceride levels. In the model predicting HDL cholesterol, only female sex emerged as a significant predictor among the variables of interest; several control variables also contributed to the prediction (alcohol use severity: *t* = 3.310, *p* < .001; BMI: *t* = −4.686, *p* < .001). The final model with insulin as the outcome variable explained 40.3% of the variance. In the final step, only PTSD diagnosis emerged as a significant predictor of increased insulin levels.

**Table 3.** Hierarchical linear regression models predicting variance in systemic inflammation levels (hs-CRP), fasting glucose levels, triglyceride levels, HDL cholesterol levels, and serum insulin levels (*N* = 176 after the imputation).

	hs-CRP			Glucose			Triglycerides			HDL Cholesterol			Insulin		
	*R*^2^	*t*	*p*	*R*^2^	*t*	*p*	*R*^2^	*t*	*p*	*R*^2^	*t*	*p*	*R*^2^	*t*	*p*
Step 1	.242			.151			.236			.203			.345		
Age		.965	.335		1.995	.046		.952	.341		.261	.794		−2.308	.022
Education level		−.877	.381		.724	.469		.309	.757		−.168	.866		.818	.415
AUDIT		−1.269	.204		1.786	.074		−.237	.812		**3**.**031**	.**002**		−1.519	.131
Smoking status		−.270	.787		−.623	.533		−1.041	.298		−1.498	.134		2.018	.045
Medication 1		.569	.569		−.138	.891		1.536	.125		.378	.706		.283	.778
Medication 2		.196	.844		–	–		–	–		–	–		–	–
Body Mass Index		**6**.**113**	**<**.**001**		**2**.**896**	.**004**		**5**.**598**	**<**.**001**		**5**.**528**	**<**.**001**		8.699	<.001
Time difference		.448	.654		.486	.627		−.820	.412		−.810	.418		..091	.928
Step 2	.321			.152			.238			.328			.352		
Sex		**4**.**234**	**<**.**001**		.396	.692		−.481	.630		**5**.**537**	**<**.**001**		1.267	.207
Step 3	.322			.152			.238			.333			.363		
Sex		4.152	**<**.**001**		.405	.686		−.509	.611		**5**.**390**	**<**.**001**		1.072	.285
PCL-5		.396	.692		−.109	.914		.277	.782		1.021	.307		1.696	.092
Step 4	.327			.154			.251			.333			.367		
Sex		**4**.**114**	**<**.**001**		.377	.706		−.578	.564		**5**.**359**	**<**.**001**		1.029	.305
PCL-5		−.075	.940		−.366	.714		−.458	.647		.777	.437		1.125	.262
PTSD/OTSR		1.029	.304		.663	.507		1.701	.089		.360	.719		1.068	.287
Step 5	.339			.155			.252			.337			.384		
Sex		**4**.**353**	**<**.**001**		.296	.767		−.503	.615		**5**.**435**	**<**.**001**		1.294	.197
PCL-5		.260	.795		−.456	.649		−.353	.724		.926	.354		1.444	.151
PTSD/OTSR		1.287	.198		.577	.564		1.761	.078		.484	.628		1.343	.181
FA		−1.887	.059		.614	.540		−.572	.567		−.936	.349		−2.108.	.037
Step 6	.356			.164			.264			.346			.403		
Sex		**4**.**209**	**<**.**001**		.388	.698		.609	.543		**5**.**537**	**<**.**001**		1.155	.250
PCL-5		.160	.873		−.365	.715		−.456	.649		1.022	.307		1.308	.193
PTSD/OTSR		2.296	.022		−.626	.531		2.366	.018		−.745	.457		**2**.**616**	.**010**
FA		.078	.938		−.544	.587		.723	.470		−1.700	.089		.129	.897
FA × PTSD/OTSR		−1.827	.071		1.355	.176		−1.580	.114		1.453	.146		−2.302	.023
Step 7	.358			.169			.269			.347			.403		
Sex		**4**.**070**	**<**.**001**		.899	.369		.023	.982		**5**.**040**	**<**.**001**		1.087	.279
PCL-5		.160	.873		−.315	.753		−.442	.659		1.028	.304		1.313	.191
PTSD/OTSR		2.246	.025		−.676	.499		2.310	.021		−.774	.439		**2**.**596**	.**010**
FA		.034	.973		−.582	.561		.665	.506		−1.728	.084		.212	.904
FA × PTSD/OTSR		−1.183	.239		1.658	.098		−.837	.403		1.570	.116		−1.860	.065
FA × PTSD/OTSR × Sex		−.929	.353		−1.040	.299		−1.026	.305		−.622	.534		−.207	.837

Notes: Hs-CRP = high-sensitivity CRP levels; AUDIT = severity of alcohol use-related problems according to the Alcohol Use Disorder Identification Test; PCL-5 = PTSD symptom severity according to the PTSD Symptom Checklist for DSM-5; FA = sum of food addiction symptoms on the Yale Food Addiction Scale (YFAS); PTSD/OTSR = post-traumatic stress disorder or other trauma and stressor-related (i.e. criterion A + 3 out of 4 PTSD symptoms) diagnosis according to the CAPS-5(-R) interview. Medication 1 for hs-CRP model = antihistamines 2nd generation; Medication 2 for hs-CRP model = all other antihistamines; Medication 1 for HOMA-IR model = all diabetes medications; Medication 1 for Triglycerides and LDL cholesterol models = all dyslipidemia medications.

## 3. Discussion

The goal of the current study was to examine the effect of threshold and subthreshold PTSD diagnosis presence, FA symptoms severity, and the interaction of PTSD/OTSR diagnosis presence and FA symptoms severity on inflammation, insulin, and select metabolic syndrome (MetS) components in a non-treatment seeking community sample of adults. Additionally, we aimed to examine the effect of sex on the PTSD/OTSR × FA interaction. We found that FA symptoms severity, but not PTSD/OTSR, was associated with greater waist circumference (WC). In analyses of the biomarkers of health, we found some effects of PTSD/OTSR, but not FA. Specifically, we found that the presence of PTSD/OTSR diagnosis was a significant predictor of higher insulin levels, but no effects of PTSD/OTSRs were found with regard to glucose, triglycerides, HDLchol or CRP. Thus, in our study, PTSD/OTSR was not associated with the MetS components (i.e. glucose, triglycerides, HDLchol and WC) or systemic inflammation but was associated with elevated insulin levels. Additionally, we found that male sex was a significant predictor of greater WC, while female sex was a significant predictor of systemic inflammation. No other sex effects were found nor were any interactive effects found.

The current study results regarding FA are somewhat consistent with past literature. Studies to date have generally found that greater FA symptom severity tends to be associated with indicators of higher body mass, such as BMI (Meule & Gearhardt, 2019; Schulte & Gearhardt, 2018) or WC (Şengör & Gezer, 2019), its association with objective health indices has been less clear (Kiyici et al., 2020). Since our sample consisted of individuals recruited from the general population, the finding of greater WC with more FA symptoms is consistent with that of Şengör and colleagues, who recruited their sample from a general (non-clinical) student population. However, this differs from studies conducted on individuals with obesity (Kiyici et al., 2020) and those with type 2 diabetes (Nicolau et al., 2020), which did not find differences in WC between groups with and without FA. It is possible that a wider range of BMI present in the general populations (in our sample the range was nearly 30) allowed for detecting the covariation between BMI and FA symptom severity better than in a medically clinical population. The significant effect of male sex on greater WC is consistent with Polish population trends wherein men more frequently experience obesity compared to women (GUS, 2016). The lack of association between FA and biomarkers of health adds to the already mixed findings in this area and suggests that despite dysregulated eating patterns that characterize FA, it does not contribute to the poorer physical health above and beyond obesity itself.

In our sample, threshold and subthreshold PTSD diagnosis was not associated with MetS components or systemic inflammation, which is contrary to the past body of research (Michopoulos et al., 2017; Nilaweera et al., 2023; Rosenbaum et al., 2015). It was however associated with higher insulin levels, consistent with past research (Michopoulos et al., 2016). Our sample consisted of non-treatment-seeking community participants – and while we oversampled individuals who obtained an elevated score on the PTSD Symptom Checklist for DSM-5 (PCL-5) (Blevins et al., 2015), the average severity of reported symptoms in the PTSD/OTSR diagnosis group was moderate (score of 44 out of possible 80, with a cutoff for possible diagnosis at 32). Therefore, it is possible that the metabolic dysfunction associated with MetS is more likely to be present in clinical treatment-seeking populations. In our sample, neither PTSD/OSTR diagnosis nor FA status was associated with increased CRP levels. It is important to note, that the average CRP levels in this sample would be considered within normal range (<3 mg/L is considered normal level in healthy persons) (Windgassen et al., 2011). Past studies have shown that PTSD severity correlates with CRP levels (Fonkoue et al., 2020; Michopoulos et al., 2015). Therefore, our sample may represent individuals with less chronic and/or less severe PTSD than other samples studied. Given that increased inflammation is considered a mechanism by which trauma exposure leads to metabolic dysfunction (Michopoulos et al., 2016), the low levels of CRP may also account for lack of findings regarding MetS components. Female sex was associated with increased inflammation, consistent with past studies that focus on sex differences in immunological responses (Klein & Flanagan, 2016).

In our sample, PTSD/OTSR diagnosis was associated with increased insulin levels, but not increased glucose. Work in children, adolescents, and animal models suggests that excess secretion of insulin by the pancreas downregulates glucose levels, contributing to the maintenance of normal levels of serum glucose (Rachdaoui, 2020; Weiss et al., 2006). However, chronic hyperinsulinemia is both a marker and a cause of insulin resistance. Moreover, prolonged excess insulin level is a predictor of adverse cardiovascular events (Kumar et al., 2019). This may be another piece of evidence to reflect the non-clinical nature of sample – elevated insulin associated with PTSD/OTSR diagnosis in this sample may be a harbinger of future metabolic dysfunction but currently is not associated with MetS.

Several limitations should be acknowledged. Participants attended phlebotomy sessions at commercial laboratories instead of research facilities. Due to the nature of the laboratories, all assays were analysed only once. As such, there is a possibility of greater error and variability in laboratory results. Additionally, despite the fact that participants were instructed to attend the phlebotomy session in the morning and in a fasted state, time of day and other variables that might influence biomarkers (e.g. physical activity, fasting adherence) were not controlled, thus potentially contributing to greater variability in data. There is also a possibility of unreported medication use influencing the results. Another limitation was the fact that 23 participants were excluded from the analyses due to incomplete clinical interview data. Methodologically, it was the most conservative course of action as, despite the fact that they did not meet criteria for PTSD diagnosis, it was impossible to determine whether they met criteria for OTSR due to the discontinuation rule on the CAPS. Given that the Polish population is rather ethnically homogenous and that our sample was recruited from a particular region in Poland, another limitation of this study is potentially limited generalizability of the current findings to individuals from other cultural and ethnic backgrounds. Future studies should prioritize examining these associations in more diverse samples.

The study also has several strengths. First, the sex distribution is approximately equal allowing for more robust analyses of the effects of sex. Second, the presence of the PTSD/OTSR diagnosis was determined using a structured clinical interview considered a gold standard for diagnosing PTSD. Therefore, the confidence of the actual presence of the diagnosis is far greater than using a cut-off score on a self-report measure.

This is the first study to examine the associations between food addiction and CRP as well as glucose levels in the general population. The lack of association between FA and objective indices of health in the general population adds to the already existing similar findings. However, addictive-like eating patterns appear to be an important target in addressing increased body mass and abdominal obesity. Our findings suggest that in a non-treatment-seeking population, the health of individuals with PTSD/OTSR is not yet markedly deterioriated (as their average lab results were still within normal range) but that they may be showing signs of potentially worsening health with higher insulin levels compared to those without PTSD/OTSR. This suggests that early detection and treatment of PTSD might be a target for improving overall health. Particularly in individuals whose immunological and metabolic indices are not yet in the clinical range, intervening at the level of PTSD symptoms may be a strategy for preventing future metabolic dysfunction. However, this is an empirical question and longitudinal studies are needed to test this hypothesis.

## Data Availability

Data associated with this project are available on the OSF server at https://osf.io/vb63x/; 10.17605/OSF.IO/VB63X.
